# Supplementary material for: The Immune System Response to Porphyromonas gingivalis in Neurological Diseases
Source: Microorganisms. 2023 Oct 13;11(10):2555. doi: 10.3390/microorganisms11102555 (PMC10609495; doi:10.3390/microorganisms11102555)
Supplement: Supplementary file 1 [file microorganisms-11-02555-s001.zip › microorganisms-2561045-supplementary.pdf]

Table S1. STROBE Statement—checklist of items that should be included in reports of observational studies.

	Item No.	Recommendation	Page No.	Relevant text from manuscript
**Title and abstract**	1	(*a*) Indicate the study's design with a commonly used term in the title or the abstract	1	case-control study
		(*b*) Provide in the abstract an informative and balanced summary of what was done and what was found	1	inadequate response of the immune system of the N-DEG group in producing anti-Pg antibodies
**Introduction**				
Background/rationale	2	Explain the scientific background and rationale for the investigation being reported	2,3	The presence of serum antibodies to major periodontal pathogens has been associated with Alzheimer's Disease. Anti-Pg antibodies were also detected on neurological patients' sera by enzyme-linked immunosorbent assays we evaluated the immune system's response to Pg abundance in the oral cavity in patients affected by pathologies in the CNS.
Objectives	3	State specific objectives, including any prespecified hypotheses	3	anti-Pg antibodies in the serum and Pg abundance in the oral cavity were quantified in patients with neurodegenerative diseases compared to healthy controls (HC) and patients affected by acute or chronic neurological diseases

**Methods**				
Study design	4	Present key elements of study design early in the paper	4	Figure 1. Flow chart of the study cohort.
Setting	5	Describe the setting, locations, and relevant dates, including periods of recruitment, exposure, follow-up, and data collection	3	HC free from neurological diseases were recruited from a list of HC. In contrast, from May 2020 to July 2022, all patients were enrolled in the Neurology Clinic of "SS Annunziata" Hospital of Chieti.
Participants	6	(*a*) *Cohort study*—Give the eligibility criteria, and the sources and methods of selection of participants. Describe methods of follow-up<br>*Case-control study*—Give the eligibility criteria, and the sources and methods of case ascertainment and control selection. Give the rationale for the choice of cases and controls<br>*Cross-sectional study*—Give the eligibility criteria, and the sources and methods of selection of participants	3	Individuals under antibiotic therapy or using daily chlorhexidine mouthwash within the last 3 months .were excluded from the study. In addition, 5 patients were excluded for concomitant tumor
		(*b*) *Cohort study*—For matched studies, give matching criteria and number of exposed and unexposed<br>*Case-control study*—For matched studies, give matching criteria and the number of controls per case	3, 4	the eligibility criteria were evaluated in 30 HC and 90 neurological patients, which could be subdivided into three groups, obtaining 1:1 matching among groups.<br>Figure 1
Variables	7	Clearly define all outcomes, exposures, predictors, potential confounders, and effect modifiers. Give diagnostic criteria, if applicable	3	tongue biofilm was used to quantify Pg abundance and blood sample was used to quantify anti-Pg antibody levels.
Data sources/ measurement	8*	For each variable of interest, give sources of data and details of methods of assessment (measurement). Describe comparability of assessment methods if there is more than one group	5	Bacterial DNA quantification on brushing (Paragrph)<br>Antibody assay on serum

				(Paragraph)
Bias	9	Describe any efforts to address potential sources of bias	5	Spearman's correlation tests were also performed separately for each group to evaluate the effect of age and sex
Study size	10	Explain how the study size was arrived at	3	In the same condition, the statistical power could reach 80% with $\alpha = 0.05$, if the sample size of 33 had been considered for each group. In addition, considering also the HC group, we estimated that with a low percentage increase of 2.4% in the ratio values, we could obtain a statistical power of 80% and α=0.05 with a sample size of 30 for each group.

Quantitative variables	11	Explain how quantitative variables were handled in the analyses. If applicable, describe which groupings were chosen and why	7	Non-parametric Kruskal-Wallis tests were performed to compare Pg bacteria and antibody quantities among groups. Specifically, logarithmic transformations were applied to Pg abundance value, anti-Pg antibodies quantity, and the ratio between anti-Pg and Pg to respect the homogeneity of the variance among groups
Statistical methods	12	(*a*) Describe all statistical methods, including those used to control for confounding	5	Data were compared among groups using non-parametric Kruskal-Wallis tests. Levene's

				test was used to test the homogeneity of the variance among groups.
		(*b*) Describe any methods used to examine subgroups and interactions	5	Post-hoc test was applied for the pairwise comparisons.
		(*c*) Explain how missing data were addressed	6	Due to the low number of participants which completed the dental visit, statistical comparisons among groups were not performed on other oral indices.
		(*d*) *Cohort study*—If applicable, explain how loss to follow-up was addressed<br>*Case-control study*—If applicable, explain how matching of cases and controls was addressed<br>*Cross-sectional study*—If applicable, describe analytical methods taking account of sampling strategy	NA	
		(*e*) Describe any sensitivity analyses	NA	
**Results**				
Participants	13*	(a) Report numbers of individuals at each stage of study—eg numbers potentially eligible, examined for eligibility, confirmed eligible, included in the study, completing follow-up, and analysed	4	Figure 1
		(b) Give reasons for non-participation at each stage	4	Figure 1
		(c) Consider use of a flow diagram	4	Figure 1
Descriptive data	14*	(a) Give characteristics of study participants (eg demographic, clinical, social) and information on exposures and potential confounders	6	Table 1
		(b) Indicate number of participants with missing data for each variable of interest	6	Table 1
		(c) *Cohort study*—Summarise follow-up time (eg, average and total amount)		
Outcome data	15*	*Cohort study*—Report numbers of outcome events or summary measures over time	NA	
		Case-control study—Report numbers in each exposure category, or summary measures of exposure	NA	
		Cross-sectional study—Report numbers of outcome events or summary measures	NA	
Main results	16	(*a*) Give unadjusted estimates and, if applicable, confounder-adjusted estimates and their precision (eg, 95% confidence interval). Make clear which confounders were adjusted for and why they were included	8	Figure 3

		(*b*) Report category boundaries when continuous variables were categorized	NA	
		(*c*) If relevant, consider translating estimates of relative risk into absolute risk for a meaningful time period	NA	
Other analyses	17	Report other analyses done—eg analyses of subgroups and interactions, and sensitivity analyses	7	No significant correlation was found between the abundance of Pg and smoking ($p = 0.35$), or prior smoking ($p = 0.99$), or teeth number ($p = 0.44$), or number of fixed prostheses ($p = 0.54$), or number of removable dentures ($p = 0.22$). No significant correlation was found between anti-Pg antibodies quantity and age ($p = 0.90$) or sex ($p = 0.07$), or smoking ($p = 0.54$), or former smoking ($p = 0.53$).
**Discussion**				
Key results	18	Summarise key results with reference to study objectives	9	This study showed that the Pg abundance in the oral cavity was higher in people with chronic neurological conditions than in HC or people affected by an acute neurological condition. Previous studies showed that oral microbiota composition originating from buccal and sublingual mucosa and salivary samples differed between healthy subjects and PD patients [57-60].
Limitations	19	Discuss limitations of the study, taking into account sources of potential bias or imprecision. Discuss both direction and magnitude of any potential bias	10	The main limitation of our study is the relatively small sample size and the heterogeneity of the

				patients' groups.
Interpretation	20	Give a cautious overall interpretation of results considering objectives, limitations, multiplicity of analyses, results from similar studies, and other relevant evidence	10	Future follow-up studies could monitor the microbiota profile and the immune response over time and at different stages of the disease to investigate the influence of periodontopathogen bacteria on the onset and progression of neurological and neurodegenerative diseases.
Generalisability	21	Discuss the generalisability (external validity) of the study results	10	we could not draw definitive conclusions on this oral bacterium's influence and the altered immune response on the pathogenesis of neurodegenerative diseases.
**Other information**				
Funding	22	Give the source of funding and the role of the funders for the present study and, if applicable, for the original study on which the present article is based	10	This research received no external funding.

*Give information separately for cases and controls in case-control studies and, if applicable, for exposed and unexposed groups in cohort and cross-sectional studies.

Note: An Explanation and Elaboration article discusses each checklist item and gives methodological background and published examples of transparent reporting. The STROBE checklist is best used in conjunction with this article (freely available on the Web sites of PLoS Medicine at http://www.plosmedicine.org/, Annals of Internal Medicine at http://www.annals.org/, and Epidemiology at http://www.epidem.com/ all published simultaneously on October 16, 2007). Information on the STROBE Initiative is available at www.strobe-statement.org.
